# Automated compilation of Urdu poetry handwritten image datasets for optical character recognition

**DOI:** 10.1016/j.mex.2024.103130

**Published:** 2024-12-21

**Authors:** Irtaza Ijaz, Abdallah Namoun, Nasser Aljohani, Meshari Huwaytim Alanazi, Mohammad N. Alanazi, Junaid Shuja, Mohammad Ali Humayun

**Affiliations:** a Computer Science Department, Information Technology University of Punjab, Lahore, Pakistan; b AI Center, Faculty of Computer and Information Systems, Islamic University of Madinah, Madinah 42351, Saudi Arabia; c Computer Science Department, College of Sciences, Northern Border University, Arar 91431, Saudi Arabia; d College of Computer and Information Sciences, Imam Mohammad Ibn Saud Islamic University (IMSIU), Riyadh 13318, Saudi Arabia; e Department of Computer Science, Southeast Missouri State University, Cape Girardeau, MO 63701, United States

**Keywords:** Optical character recognition, Urdu poetry, Automated data curation, Text processing, Deep neural networks, Handwritten documents, Character recognition systems, Automated image segmentation and labeling for Urdu Poetry OCR dataset

## Abstract

Optical character recognition (OCR) is vital in digitizing printed data into a digital format, which can be conveniently used for various purposes. A significant amount of work has been done in OCR for well-resourced languages like English. However, languages like Urdu, spoken by a large community, face limitations in OCR due to a lack of resources and the complexity and diversity of handwritten scripts. One of the major hindrances in the development of OCR for low-resource languages like Urdu is the lack of extensive datasets. However, such datasets can be obtained from old handwritten books with reference text available online. This study presents a method to leverage this resource and automatically process Urdu handwritten poetry books with corresponding scripts available online. The images are segmented at the sentence level using automated neighborhood-connected component analysis, followed by manual adjustment. Corresponding Unicode text for each image are obtained by web scraping followed by text similarity analysis. A sample dataset collected comprises purely handwritten Urdu text images for Urdu poetry by Mirza Ghalib and Allama Iqbal, arguably the two most influential poets in Urdu. The dataset comprises 2888 images with Unicode transcriptions from poetry by Mirza Ghalib and Allama Iqbal.

• The method automates OCR dataset creation by segmenting handwritten text images and scraping corresponding text from the web for alignment.
• Handwritten images are segmented into sentences using a resource-efficient Neighborhood Component Analysis approach.
• Possible text samples are scraped from the web, and the corresponding labels are aligned with images based on the minimum edit distance between the scraped text and the predictions by an OCR engine.

## Graphical abstract

**Figure:**
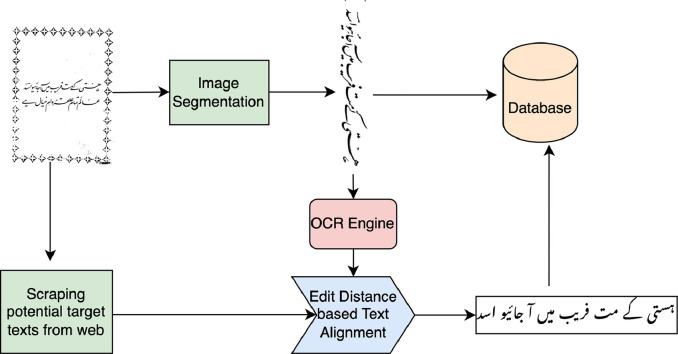

Specifications table

**Table:** 

Subject area:	Computer Science
More specific subject area:	Computer Vision and Pattern Recognition
Name of your method:	Automated image segmentation and labeling for Urdu Poetry OCR dataset
Name and reference of original method:	None
Resource availability:	The code presented in this paper is available at:
	https://github.com/mohammadalihumayun/OCRdataset
	The sample dataset is publicly available on the repository:
	https://data.mendeley.com/datasets/gr5vytfdw7/1

## Background

Optical Character Recognition (OCR) enables handwritten or printed text to be automatically converted into machine-encoded text. OCR's applications range from basic document digitization to helping the blind read text with assistive devices. OCR has a wide range of applications, ranging from board and car plate recognition to the digitization and preservation of historic documents for easy editing, searching, and translation.

>200 million people speak Urdu, a significant language of South Asia, mostly in Pakistan and India. Due to its rich vocabulary derived from Persian, Arabic, and Turkic languages, Urdu is a significant cultural and historical language in the area. Urdu's literary and cultural relevance highlights the need for reliable OCR systems to digitally preserve, distribute, and evaluate Urdu literature.

Nevertheless, creating an OCR system for Urdu is a difficult process. A major challenge is the scarcity of comprehensive datasets, which are necessary for developing precise recognition algorithms. In contrast to the majority of Western languages, Urdu has a shortage of digital text data, making it difficult to develop efficient OCR technology. Additionally, the intricate calligraphic writing style of the Urdu script presents particular difficulties. Urdu, although similar to Arabic and Persian, has significantly more glyphs and ligatures and fewer resources, making it even more challenging [1,2].

Recently, researchers have manually collected large handwritten Urdu OCR datasets and successfully trained deep learning models to achieve good results. However, the models still struggle to generalize across multiple domains and genres within Urdu handwritten texts [3,4].

This work seeks to improve the digital accessibility of Urdu literature by presenting an automated method for collecting dataset of Urdu handwritten poetry. The method involves automatically extracting images from scanned copies of handwritten old books, then employing neighborhood component analysis [5] to extract sentence-level segmented images. Finally, web scraping, existing OCR models and text similarity measures are used to fetch the corresponding Unicode text labels. Although recently deep learning-based models like YOLO [6] have achieved significant results for object detection and can be employed for sentence segmentation, they are resource and data-intensive. In contrast, this study focuses on the same task using less resource-intensive methods.

## Method details

Our technique utilizes an automated data processing pipeline to get segmented line images from old scanned books and fetch corresponding labels from web. The existing and datasets are either collected manually by handwriting and taking images of the text already available in Unicode or by manually typing in the text labels for available handwritten images. The presented method introduces a pipeline that not only automatically extracts images for handwritten text but also gets the corresponding labels from web sources automatically. Thus, the unique contributions of the dataset can be listed are as follows:

1. A method is developed to automatically segment images with handwritten sentences
2. The method automatically aligns the images with the corresponding text labels scraped from web
3. A sample dataset has been collected for Urdu poetry, comprising works from the two most famous Urdu poets.
4. The dataset, along with the code for automatically collecting the dataset have been made public [7,8].

Initially, the PDF pages are split into individual images then image processing is utilized to segment the images into individual text lines. The images were extracted from PDF copies of scanned handwritten books by two famous Urdu poets using Python libraries for PDF segmentation.

Then images were segmented into text lines using the Python OpenCV library employing the Neighborhood Component Analysis algorithm. Particularly the Neighborhood Connected Component Analysis is used to split the page into text line segments [5]. The algorithm involves several key steps. First, it uses an eight-way Connected Component Labeling algorithm to label and identify components. It calculates the average heights and widths of these components and ignores those that are significantly smaller or larger during preprocessing. Large components that might cause line merging are split. Each component's neighborhood is defined based on the average height and width to find other components on the same line. Initially ignored small components are reconsidered in post-processing and assigned to the appropriate text lines, ensuring accurate text line formation from scanned document images.

**Fig. 1:**
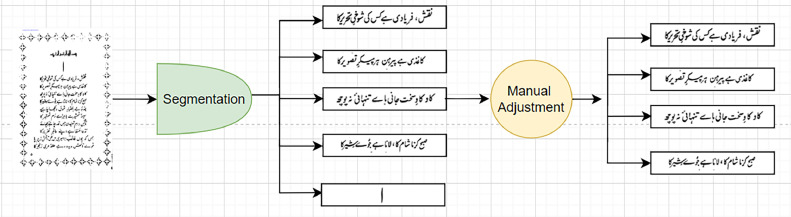
Sentence images segmentation pipeline.

The data then underwent some manual trimming, and cleaning, to ensure the image isn't hampered by noise, markings, or extraneous text. Every divided line is stored as a distinct image file and is titled using the page and line number segmented from the page (e.g., page_1_line_1.png) although not each segmented line by the algorithm contained text hence the line numbers are not contiguous. Fig. 1 depicts an explanation for the segmentation and manual adjustment process for each image.

Finally, the annotations for the images were found by web scraping and employing available OCR models using Python libraries besides manual intervention for typing. Each of the images was translated to text using the best Urdu OCR model available i.e. UTRNET [3,9]. UTRNet is a CNN-RNN hybrid architecture that has achieved state-of-the-art results for Urdu OCR after being trained on two datasets, UTRSet-Real and UTRSet-Synth.

In order to look up the corresponding text from the Unicode text bank available for the poets online [10], we computed the Character Error Rate (CER) with reference to each of the available Unicode references for the poet and selected the nearest one. CER refers to the percentage of characters that the OCR model improperly recognizes. It is computed by adding the number of character substitutions, deletions, and insertions required to match the predicted text with the reference text and dividing by the number of characters in the reference text. Consequently, reference text with minimum CER was selected as the label for each image.

## Method validation

**Fig. 2:**
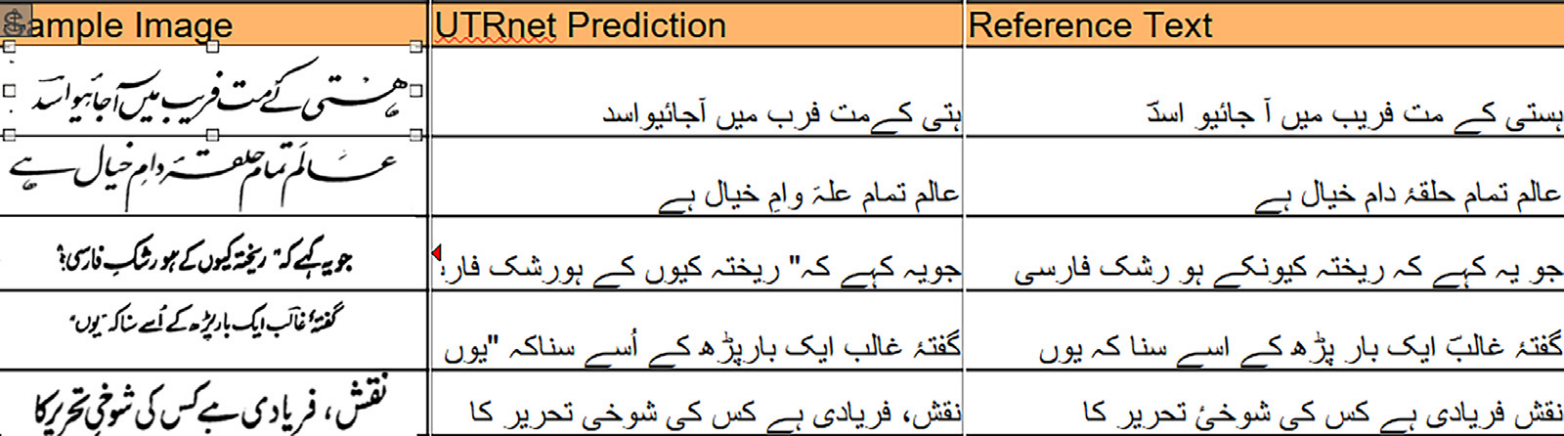
Automated reference text lookup for raw predictions.

All the automatically extracted texts were then manually verified and rectified to be representative of the text within the image to be saved as the Unicode annotations. The Unicode text annotations for the images were saved within the annotations.csv file with the first column indicating the image file name and the second column indicating the Unicode text for the file. Fig. 2 illustrates few examples of predicted texts and adjusted reference texts as final annotations.

**Fig. 3:**
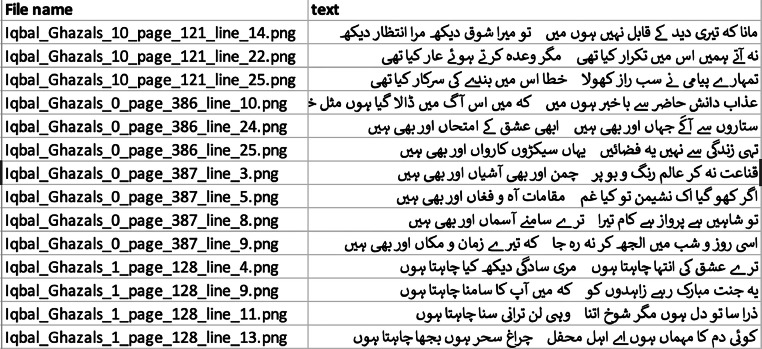
Final dataset structure.

The final compiled Urdu Poetry dataset comprises 2888 images of Urdu poetry lines by Mirza Ghalib and Allama Iqbal. Every image is a segmented single-line photo and is accompanied by corresponding Unicode text within an annotations file for the dataset. The poetry lines are stored as distinct image files named using the page and line numbers from the scanned book (e.g., Ghalib_page_1_line_1.png, Iqbal_Nazms_0_page_1_line_1.png, Iqbal_Ghazals_0_page_1_line_1.png). The text annotations are added against these file names within an annotations.csv file accompanied by the dataset. However, it is noteworthy that the line numbers represent the segmented line contours from the book, and not each contour necessarily had a text line hence the line numbers within the file names in the dataset aren't sequential and might have some missing number. Fig. 2 shows a screenshot of the annotations file. Fig. 3

Finally, CER has been computed for the complete dataset using UTRNET without any finetuning. The trained model achieved a notably lower Character Error Rate (CER) of 11.13 % and a Word Error Rate (WER) of 46.22 % which is quite impressive as the model wasn't finetuned on this dataset and hence hadn't seen any example of the images.

## Limitations

The method presents an automated process to segment sentence-level images from handwritten Urdu books and transcribe the corresponding reference text from online resources. Hence the method is effective only for older books written in handwritten form but with corresponding text already available online. Moreover, the segmentation at the sentence level assumes that the sentences are already separated in the images, which works primarily for poetry books. Therefore, this method is effective only for old poetry books with reference texts available online. The sample dataset compiled by this study is limited to 2888 handwritten lines of Urdu poetry by Mirza Ghalib and Allama Iqbal. While this dataset provides insightful information, it currently focuses on just two poets from different eras. Furthermore, after segmentation, there is some requirement for manually intervention for data organization.

The main focus of the study is how to efficiently generate and clean such a dataset to improve Urdu text recognition. However, the dataset might not accurately depict every variation of Urdu handwriting. The methodology makes use of data cleansing and collection methods designed especially for handwritten Urdu. This work aims to aid in creating better Urdu text recognition systems by addressing the difficulties associated with the task. To eliminate human interaction in future research, more sophisticated cleaning techniques and considerations for Urdu text recognition beyond handwriting would be necessary.

Although complex transformer-based deep learning architectures are already available, they can only be utilized to their full potential with large-scale datasets. The method presented can be of great value for enhancing research in Urdu OCR by automating the collection of handwritten image datasets with corresponding labels. The large-scale dataset collected using the method can significantly improve the results of Urdu OCR.

As future work, the algorithm needs to incorporate sentence boundary detection to for collecting non-poetry datasets. Moreover, the neighborhood-connected component analysis segmentation technique employed by this method can be compared in terms of effectiveness and resource efficiency with modern deep learning segmentation techniques such as YOLOv8. Finally, the method can be applied to collect a dataset from a wider range of diverse sources.

## Ethics statements

The authors have read and followed the ethical requirements for publication in MethodsX and confirm that the current work does not involve human subjects, animal experiments, or any data collected from social media platforms.

## CRediT authorship contribution statement

**Abdallah Namoun, Mohammad Ali Humayun:** Conceptualization, Project administration, Supervision, Writing. **Irtaza Ijaz, Junaid Shuja:** Methodology, Data Curation, Writing. **Nasser Aljohani, Meshari Huwaytim Alanazi, Mohammad N. Alanazi:** Validation, Formal analysis

## Declaration of competing interests

The authors declare that they have no known competing financial interests or personal relationships that could have appeared to influence the work reported in this paper.

## Data Availability

The code and dataset are available publicly and links have been added within the manuscript
